# Exon 3 of the *NUMB* Gene Emerged in the Chordate Lineage Coopting the NUMB Protein to the Regulation of MDM2

**DOI:** 10.1534/g3.119.400494

**Published:** 2019-08-26

**Authors:** Stefano Confalonieri, Ivan Nicola Colaluca, Andrea Basile, Salvatore Pece, Pier Paolo Di Fiore

**Affiliations:** * IEO, European Institute of Oncology IRCCS, Milan, Italy and; † Department of Oncology and Hemato-Oncology, Università degli Studi di Milano, 20142 Milan, Italy

**Keywords:** NUMB, MDM2, exon gain, chordate evolution, PTB domain

## Abstract

MDM2 regulates a variety of cellular processes through its dual protein:protein interaction and ubiquitin ligase activities. One major function of MDM2 is to bind and ubiquitinate P53, thereby regulating its proteasomal degradation. This function is in turn controlled by the cell fate determinant NUMB, which binds to and inhibits MDM2 via a short stretch of 11 amino acids, contained in its phosphotyrosine-binding (PTB) domain, encoded by exon 3 of the *NUMB* gene. The NUMB-MDM2-P53 circuitry is relevant to the specification of the stem cell fate and its subversion has been shown to be causal in breast cancer leading to the emergence of cancer stem cells. While extensive work on the evolutionary aspects of the MDM2/P53 circuitry has provided hints as to how these two proteins have evolved together to maintain conserved and linked functions, little is known about the evolution of the *NUMB* gene and, in particular, how it developed the ability to regulate MDM2 function. Here, we show that *NUMB* is a metazoan gene, which acquired exon 3 in the common ancestor of the Chordate lineage, first being present in the Cephalochordate and Tunicate subphyla, but absent in invertebrates. We provide experimental evidence showing that since its emergence, exon 3 conferred to the PTB domain of NUMB the ability to bind and to regulate MDM2 functions.

The *NUMB* family of genes in vertebrates comprises two genes, *NUMB* and *NUMBL* (Gulino *et al.* 2010; Pece *et al.* 2011), the former being the best characterized. The NUMB protein is involved in diverse cellular phenotypes, including cell fate determination and maintenance of stem cell compartments, regulation of cell polarity and adhesion, and migration (Nishimura and Kaibuchi 2007; Bresciani *et al.* 2010; Pece *et al.* 2011; Sato *et al.* 2011; Zobel *et al.* 2018). Four major isoforms of NUMB have been described in mammals, produced by alternative splicing of two exons (Ex3 and Ex9), which mediate distinct cellular and developmental functions (Dho *et al.* 1999; Bechara *et al.* 2013; Rajendran *et al.* 2016). In particular, Ex3 encodes for a short stretch of 11 amino acids embedded in the phosphotyrosine-binding (PTB) domain, located at the N-terminus of the protein, thus yielding two alternative versions of the domain: an Ex3-containing and an Ex3-devoid PTB domain (henceforth, PTB-long and PTB-short, respectively) (Dho *et al.* 1999). The comparative structural features of these two PTB versions have recently been resolved (Colaluca *et al.* 2018). In contrast, NUMBL does not contain sequences resembling the alternatively spliced Ex3 or Ex9.

At the biological level, NUMB and NUMBL display both redundant and non-redundant functions. *NUMBL* KO mice do not display any overt phenotype, with the exception of a reduction in female fertility (Petersen *et al.* 2002). Conversely, *NUMB* KO mice are embryonic lethal and the phenotype is worsened in double NUMB/NUMBL KOs (Petersen *et al.* 2002). This might be due, in part, to the restricted pattern of expression of NUMBL (developing nervous system) *vs.* the more abundant and ubiquitous distribution of NUMB (Zhong *et al.* 1997). Biological differences between the two proteins might also underscore their different impact on a number of signaling pathways: an issue presently unclear given the scarce characterization of NUMBL at the cellular and biochemical level *vs.* the wealth of evidence available for NUMB. A schematic representation of human NUMB and NUMBL, with their relevant protein domains and splicing patterns is shown in Figure 1A.

**Figure 1:**
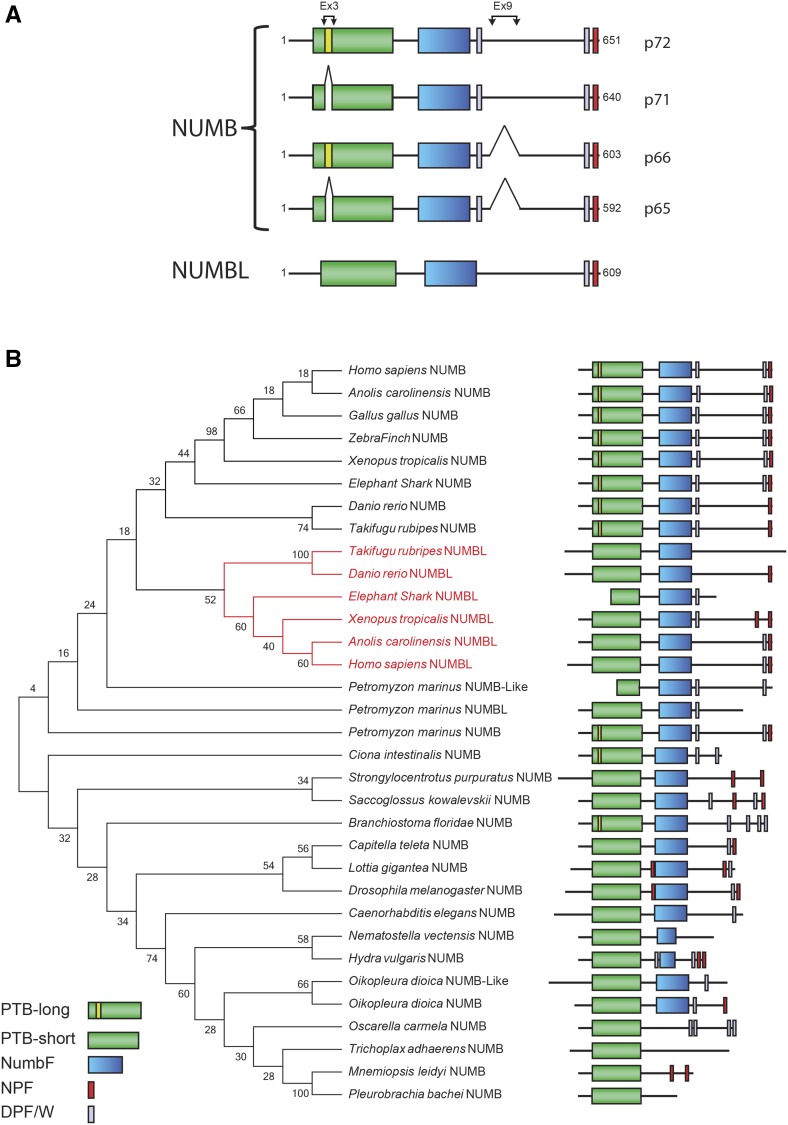
Evolution of the NUMB protein family. **A**. Structure and splicing isoforms of human NUMB and NUMBL proteins. PTB domains are in green with the portion encoded by Ex3 highlighted in yellow, the NumbF domain is in blue, NPF and DPF/W motifs, responsible for binding to endocytic proteins, are in red and gray respectively. The positions of Ex3 and Ex9 coded inserts are indicated. **B**. Minimum Evolution Phylogenetic tree of the NUMB Protein Family. The percentage of replicate trees in which the associated taxa clustered together in the bootstrap test are shown next to the branches. Protein domains and motifs are depicted as in (A). Branches in red show NUMBL proteins. Only the longest isoform in each organism is shown.

At the biochemical level, NUMB is involved in the regulation of several signaling circuitries, including Notch-, Hedgehog- and P53-dependent ones (Pece *et al.* 2004; Anderson *et al.* 2005; Di Marcotullio *et al.* 2006; Colaluca *et al.* 2008; Liu *et al.* 2011b; Pece *et al.* 2011; Tosoni *et al.* 2015; Guo *et al.* 2017). Many of the functions of NUMB have been directly or indirectly ascribed to its ability to regulate endocytic circuitries (Santolini *et al.* 2000; Sato *et al.* 2011), with particular emphasis on internalization, recycling routes and cell migration (Zobel *et al.* 2018). These endocytic properties of NUMB are evidenced by its numerous interactions with components of the endocytic machinery, including the endocytic adaptor AP2 (Santolini *et al.* 2000; Hutterer and Knoblich 2005; Song and Lu 2012), endocytic accessory proteins harboring EH domains (NUMB is an EH-binding protein) (Salcini *et al.* 1997; Smith *et al.* 2004), and others (Krieger *et al.* 2013; Wei *et al.* 2018). While these proteins bind to different regions of NUMB, their binding is apparently independent of the NUMB isoform, possibly attesting to an ancestral endocytic function(s) of NUMB that predates the appearance of its alternatively spliced isoforms.

In contrast, the binding of NUMB to the E3 ubiquitin ligase, MDM2, which results in inhibition of MDM2 enzymatic activity, is isoform-specific, depending on the presence of Ex3 in the PTB domain (Juven-Gershon *et al.* 1998; Colaluca *et al.* 2008; Colaluca *et al.* 2018). The net result of NUMB binding to MDM2 is stabilization of P53 due to inhibition of MDM2-dependent ubiquitination and ensuing proteasomal degradation of P53 (Colaluca *et al.* 2008). This is relevant to breast cancer in which loss of NUMB expression results in reduction of P53 levels and P53-mediated responses, including sensitivity to genotoxic drugs and maintenance of homeostasis in the stem cell compartment (Pece *et al.* 2004; Tosoni *et al.* 2015; Tosoni *et al.* 2017). Surprisingly, this function of NUMB is entirely controlled by the Ex3-coded sequence, as demonstrated by our recent study showing that this short fragment is both necessary and sufficient for the interaction with MDM2 and for the regulation of P53 stability (Colaluca *et al.* 2018). This result raises interesting questions about the evolution of NUMB, and in particular of the Ex3-encoded sequence, in relationship to MDM2.

The first *MDM-like* gene appeared in Placozoa, where it is able to bind to and to regulate the levels of P53 (Siau *et al.* 2016; Åberg *et al.* 2017). This ancestral gene duplicated to generate *MDM2* and *MDM4* in vertebrates (Momand *et al.* 2011; Åberg *et al.* 2017), acquiring also several P53-unrelated functions (Carrillo *et al.* 2015; Saadatzadeh *et al.* 2017). The *NUMB* gene has been hypothesized to be a metazoan invention (Gazave *et al.* 2009); however, nothing is known about the appearance of the Ex3-containing NUMB isoforms (Dho *et al.* 1999). Since the P53/MDM2 signaling pathway dates back to early metazoan time and has then tightly co-evolved (Momand *et al.* 2011; Lane and Verma 2012; Åberg *et al.* 2017), it is tempting to speculate that also the *NUMB* gene followed a similar evolutionary route, as partner and regulator of MDM2 functions. Thus, important lessons could be learned by following the evolutionary trajectory of the mammalian *NUMB* Ex3, which could also be relevant to human health, since we previously demonstrated that the relative expression of Ex3-containing NUMB isoforms is clinically relevant in predicting the outcome of breast cancers (Colaluca *et al.* 2018). The present study was undertaken to investigate this issue.

## Materials and Methods

### Indentification of PTB domain-containing proteins and phylogenetic analysis of NUMB homologs

Protein, ESTs and mRNA sequence databases were downloaded from NCBI or ENSEMBL databases, from the organism specific databases (Borowiec *et al.* 2015), from Compagen (http://www.compagen.org/) or from the Joint Genome Institute (http://genome.jgi.doe.gov/) databases. Searches were carried out on 31 complete proteomes (Table S1). Each proteome was made non-redundant with CD-HIT (Li and Godzik 2006). The HMMER software version 3.1b2 (Eddy 2011) was used to search for PTB or IRS domain-containing proteins, with the SMART profile for the PTB or IRS domains. Domain annotations were obtained with the PFAM-A domain collection (Pfam 31.0 release) (Finn *et al.* 2016) (Table S3). ClustalX was used for all multiple alignments (Thompson *et al.* 2002) and visualized with Jalviev (Waterhouse *et al.* 2009). Evolutionary tree analyses were carried out using the Minimum Evolution method with MEGA7 (Kumar *et al.* 2016). The bootstrap consensus tree inferred from 50 replicates is taken to represent the evolutionary history of the taxa analyzed. Branches corresponding to partitions reproduced in less than 50% bootstrap replicates were collapsed. The percentage of replicate trees in which the associated taxa clustered together in the bootstrap test (50 replicates) are shown next to the branches. The evolutionary distances were computed using the JTT matrix-based method and are in the units of the number of amino acid substitutions per site. The ME tree was searched using the Close-Neighbor-Interchange (CNI) algorithm at a search level of 1. The Neighbor-joining algorithm was used to generate the initial tree. Exon annotations were made with the GeneWise software (Birney *et al.* 2004), using the genome and the protein databases from the same species, when available. To find possible Ex3-like sequences, if not present in the protein database, genome loci were additionally searched with GeneWise using the Human or the PTB-long domain-containing NUMB from the closest species.

### Biochemical studies and reagents

Mammalian expression vectors for P53 and MDM2 were a gift from K. Helin (Cell Biology Program, Memorial Sloan Kettering Cancer Center, New York). Mammalian expression vectors encoding various NUMB fragments were engineered in a pcDNA vector in-frame with a FLAG-tag at the C-terminus. The *Ciona* cDNAs for MDM2 and NUMB was synthesized from Proteogenix and designed on the sequences AK116170 for MDM2, and AB210594 and XM_009859573 for NUMB. NUMB-PTB point mutations (Colaluca *et al.* 2018) were generated by the QuikChange strategy (Agilent Technologies) according to the manufacturer’s instructions. The gene modifications and inserts were confirmed by DNA sequencing. Procedures for immunoblotting (IB) and immunoprecipitation (IP) were as described previously (Pece *et al.* 2004) according to standard procedures. For the *in vivo* p53 ubiquitination assay (Figure 3D), 10-cm plates of H1299 cells were transfected with 1 µg P53-coding vector, 1.3 µg MDM2-coding vector, 5 µg His6-Ub–coding vector and 5 µg NUMB-coding vector. H1299 were chosen since they are P53 null. After 48 h, ubiquitin conjugates were purified as described previously by (Colaluca *et al.* 2018). Antibodies were anti-FLAG M2-agarose affinity gel from Sigma-Aldrich, anti-Mdm2 (OP46) from EMD Millipore, anti-p53 (DO-1) from Santa Cruz Biotechnology, Inc., and anti-FLAG and anti-HA from Cell Signaling Technology, Inc.

### Data availability

Figure S1 contains the analysis of the PTB domain containing protein found in the analyzed organisms. Figure S2 contains the alignment of the NUMB and NUMBL proteins found. Figure S3 contains the alignment of vertebrate NUMB and NUMBL proteins together with the gene structure analysis. Figure S4 contains the alignment of the NumbF domain of NUMB and NUMBL proteins. Figure S5 contains the analysis of the two Oikopleura dioica NUMB genes and proteins. Figure S6 contains the analysis of the NUMB and NUMBL proteins found in the Sea and Arctic Lamprey. File S1 contains two supplementary tables and legend for all supplementary figures and tables. Table S3 is an excel file containing contains the detailed output of the HMMER search (version 3.1b1) for each organism, together with accession numbers, protein domain composition and closest homologs identified by reverse BLAST search for each identified protein.

Supplemental material available at FigShare: https://doi.org/10.25387/g3.8427110.

## Results and Discussion

### Identification of NUMB homologs in evolution

The defining characteristic of NUMB (and NUMBL) is the presence of a PTB domain. To shed light on the evolution of the Ex3-coded sequence of NUMB, which is contained in its PTB domain, we identified all proteins potentially containing a PTB domain in representative and fully sequenced metazoans (Borowiec *et al.* 2015). We included in the search, proteins containing an insulin receptor substrate-like PTB domain (IRS), which is a variant of the canonical PTB domain (Eck *et al.* 1996; Uhlik *et al.* 2005). In addition to metazoans, we analyzed the proteome of the two choanoflagellates, *Monosiga brevicollis* and *Salpingoeca rosetta*, since it has been reported that choanoflagellates, the closest known unicellular relatives of animals, contain a surprising variety and high number of tyrosine kinase and PTB domains (Manning *et al.* 2008; Pincus *et al.* 2008). Finally, we included in our analysis the amoeboid protozoan *Dictyostelium discoideum* and the plant *Arabidopsis thaliana* as controls (see Table S1 for a complete list of organisms included in this study). Each identified protein was analyzed individually for domain composition using the PFAM and SMART protein domain databases and BLASTed against all vertebrate proteins to identify the true ortholog. This method has been reported to be more reliable than using automatic annotation software, such as Ensembl and InParanoid (Liu *et al.* 2011a).

By this approach, we confirmed the identity of the sequences retrieved with the initial HMM-profile searches (obtained using the HMMER 3.1b2 software) and defined each true ortholog of all human PTB- or IRS-containing proteins (Figure S1; see also Tables S2 and S3 for protein descriptions and the detailed HMM profile search results and domain annotation). NUMB homologs were identified by a reciprocal best hits BLAST search in each organism, and by the presence of the NumbF domain (see below), and of DPF (Aspartate, Proline, Phenylalanine) and NPF (Arginine-Proline-Phenylalanine) motifs, which mediate NUMB (and NUMBL) interaction with the AP2 protein complex and EPS15, respectively (Salcini *et al.* 1997; Owen *et al.* 2000)(Figure 1A).

### Numb is a metazoan gene and is duplicated in the vertebrate lineage

*NUMB* first appeared in evolution in Metazoa (Figure 1B and Figure S2), being present as a single gene in Ctenophora, as previously reported (Gazave *et al.* 2009), with the two distinct paralogs, *NUMB* and *NUMBL*, emerging only in vertebrates. The *NUMB* and *NUMBL* genes exhibit very similar exon–intron organizations with highly conserved intron positions and phases (Figure S3). In addition to the N-terminal PTB domain, NUMB proteins display a central conserved domain (NumbF, NUMB-Family protein domain) and a C-terminal proline-rich region (PRR). The NumbF domain is a region of unknown function, present only in the NUMB family of proteins (both NUMB and NUMBL), which is well conserved among NUMB homologs (Figure S4) and has been shown to be involved in the binding to RAB7A/B and ERBB2 (Hirai *et al.* 2017), while the PRR is poorly conserved (Figure S2). *NUMB* seems to be present only in one species of Porifera, the Slime sponge *Oscarella carmela*. We found no evidence of the existence of a *NUMB* gene in three other Porifera proteomes and genomes analyzed (Table 1), possibly due to incomplete genome assembly/annotation of the relative genomes, or to selective loss of the gene, similar to what has been observed for other genes in demosponges (Riesgo *et al.* 2014). Notably, partial or complete gene loss is responsible for the lack of *MDM* and *P53*/*P63*/*P73* genes in Porifera (Åberg *et al.* 2017).

**Table 1:** Protein and transcript analysis of NUMB in evolution

	Protein Analysis			Transcript Analysis			
Species	N. of Proteins	NUMB Protein	NUMB PTB-Long Isoform	N. of cDNA	N. of ESTs or RNA-SEQ data	NUMB mRNA	NUMB PTB- Long Isoform mRNA
**Homo sapiens**	31,283	YES	YES	9,774,024	8,704,790	YES	YES
***Taeniopygia guttata (Song Bird)***	17,758	YES	YES	79,179	92,142	YES	NO
***Gallus gallus***	20,979	YES	YES	387,867	600,434	YES	YES
***Anolis carolinensis (Lizard)***	20,396	YES	YES	8,588,979	156,802	YES	YES
***Xenopus tropicalis***	22,785	YES	YES	78,985	1,271,480	YES	YES
***Fugu rubripes***	21,423	YES	N.A.^a^	97,191	566,629	NO	NO
***Danio rerio***	27,033	YES	YES	220,453	1,488,339	YES	YES
***Callorhinchus milii (Elephant shark)***	18,806	YES	YES	91,515	109,965	YES	YES
***Petromyzon marinus (Sea Lamprey)***	11,442	YES	YES	28,215	120,731	NO	NO
***Ciona intestinalis***	15,453	YES	YES	38,404	1,205,674	YES	YES
***Oikopleura dioica***	18,020	YES	NO	5,972	207,355	YES	NO
***Branchiostoma floridae***	39,596	YES	YES	30,270	334,502+ RNA-Seq	YES	YES
***S. kowalevskii (Acorn worm)***	20,381	YES	NO	85,678	202,190	YES	NO
***S. purpuratus (purple sea urchin)***	25,045	YES	NO	187,092	141,833	YES	NO
***Lottia gigantea (Owl Limpet)***	34,510	YES	NO	32,851	252,091	YES	NO
***Capitella teleta (polychaete worm)***	45,370	YES	NO	20,969	138,404	NO^b^	NO
***Caenorhabditis elegans***	20,981	YES	NO	150,848	538,795	YES	NO
***Drosophila melanogaster***	15,322	YES	NO	320,780	847,942	YES	NO
***Nematostella vectensis (sea anemone)***	44,353	YES	NO	532,252	163,314	YES	NO
***Hydra vulgaris***	17,266	YES	NO	172,012	184,731	YES	NO
***Trichoplax adhaerens***	11,520	YES	NO	14,453	11,498	NO	NO
***Amphimedon queenslandica (sponge)***	13,721	NO	NO	1,042	83,040	NO	NO
***Oscarella carmela (slime sponge)***	11,152	YES	NO	22	11,176 + RNA-Seq	YES	NO
***Xestospongia testudinaria (barrel sponge)***	21,880	NO	NO	N.A.	22,337	NO	NO
***Stylissa carteri (elephant ear sponge)***	24,925	NO	NO	N.A.	26,967	NO	NO
***Mnemiopsis leidyi (Sea Walnut)***	16,548	YES	NO	5,493	15,752 + RNA-Seq	YES	NO
***Pleurobrachia bachei (Sea gooseberry)***	19,524	YES	NO	115	298 + RNA-Seq	YES	NO
***Salpingoeca rosetta***	11,731	NO	NO	N.A.	7	N.A.	N.A.
***Monosiga Brevicollis***	9,196	NO	NO	N.A.	29,595	N.A.	N.A.
***Dictyostelium discoideum***	11,914	NO	NO	N.A.	N.A.	N.A.	N.A.

NUMB protein sequences retrieved with the initial HMM search were analyzed for the presence of a PTB-long isoform at the protein and transcript level: numbers of total sequences available are indicated. ^a^ No ESTs or mRNA sequence were found for the Fugu NUMB transcript, therefore the existence of Ex3 cannot be assessed. ^b^ An EST corresponding to the NUMB predicted transcript of *Capitella telata* is present among the ESTs of *Capitella capitata* (JGI_CAWU12746.fwd, 100% ID), suggesting the existence of a NUMB transcript in Polychaeta Worms. N.A., no sequence available for the analysis.

Most of the proteins identified were predictions from the genomes, and the existence of a gene, or of a predicted exon, does not necessarily imply that the mRNA will be expressed, or a particular exon included or excluded in the mature mRNA transcript. Thus, we investigated whether the identified NUMB proteins are actually transcribed. As summarized in Table 1, we analyzed all available transcript databases, *i.e.*, mRNAs, ESTs, TSA (Transcriptome Shotgun Assembly) or RNA-Seq data for each species; the presence of a NUMB transcript was confirmed in all species with the exception of *Fugu rubripes*, *Petromyzon marinus* (Sea Lamprey), *Capitella telata*, *Trichoplax adherens*, *Amphimedon queenslandica*, *Xestospongia testudinaria*, *Stylissa carteri*, and non-metazoan organisms as expected. An EST for the *NUMB* gene of *Capitella capitata* is present in the database, suggesting the existence of a NUMB transcript in Polychaeta.

We were able to confirm that the *NUMB* gene and transcript first appeared in metazoans, but they do not seem to be present in all species of sponges. Invertebrates contain a single *NUMB* homolog, while a *NUMBL* paralog appears only in the vertebrate lineage. As in the case of MDM2/MDM4 and P53/63/73, the invertebrate NUMB proteins are more similar to one another than to either of their vertebrate orthologs (Belyi *et al.* 2010; Tan *et al.* 2017). Indeed, in a phylogenetic analysis, only vertebrate NUMB and NUMBL proteins clearly separated into two distinct branches of the tree (Figure 1B).

Exceptions were noted in the Tunicate *Oikopleura dioica* and in Jawless Fishes, in particular:

1. In *Oikopleura dioica*, two predicted NUMB-related proteins are present belonging to two distinct genes that are both expressed, as supported by the presence of several ESTs (Figure S5). At the protein level, the two paralogs display a low level of similarity (24% identity) and they seem more similar to human NUMB than to human NUMBL (Figure S5).
2. In the Jawless fish, Sea Lamprey (*Petromyzon marinus*), three genes encoding putative *NUMB* homologs are present. All three proteins are predicted (NUMB, ENSPMAP00000001971; NUMBL, ENSPMAP00000006450; NUMB-Like, ENSPMAP00000002145), and contain a PTB and a NumbF domain, in addition to NPF and DPF motifs (Figure 1B); however, ENSPMAP00000002145 displays a partial PTB and does not possess the starting methionine (Figure S6). In order to verify whether this occurrence is peculiar to the Sea Lamprey, we searched for NUMB homologs in the Japanese Lamprey (*Lethenteron camtschaticum*) Database (http://jlampreygenome.imcb.a-star.edu.sg/) and found three proteins, with similar domains and motif content (Accession numbers: NUMB, JL4438; NUMBL, JL5974; NUMB-Like, JL2165). Notably, the NUMB-Like predicted protein is a fragment, containing a partial PTB domain, lacking the starting methionine and the NumbF domain, but yet very similar to the Sea Lamprey NUMB-Like (Figure S6). Each Lamprey orthologs cluster together, as expected, but not with the respective human ortholog (Figure 1B and Figure S6). The absence of any mRNA or EST sequences does not allow verification of whether any of the three genes are actually transcribed (Table 1).

### The PTB-Long isoform of NUMB is a chordate invention

**Figure 2:**
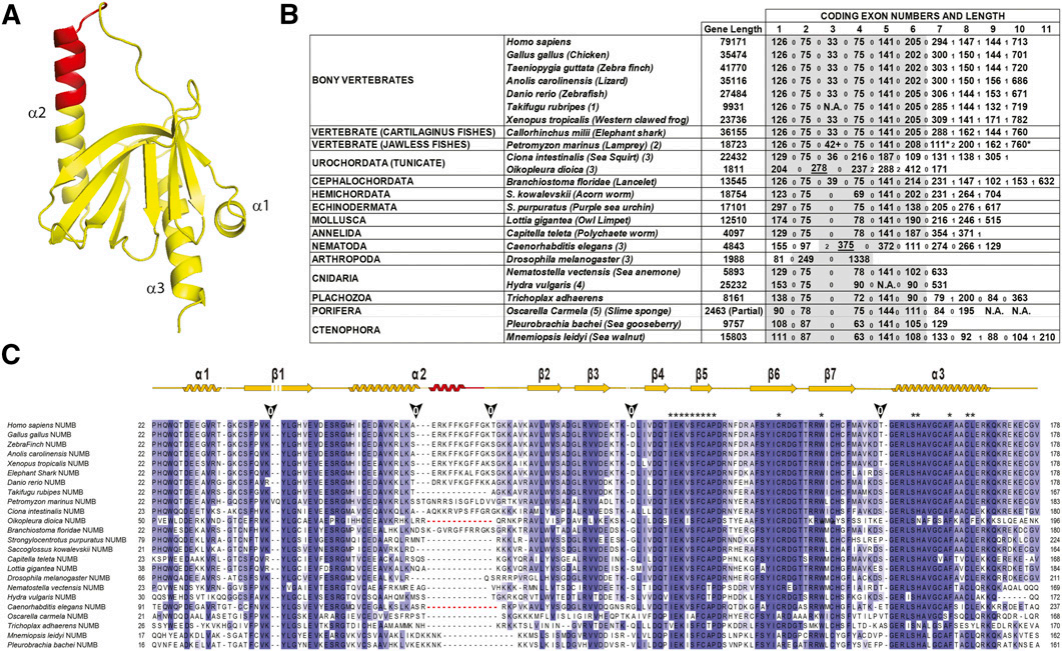
Ex3 of the human *NUMB* gene is a Chordate invention. **A**. Structure of the PTB-long domain of human NUMB (Colaluca *et al.* 2018); the Ex3 encoded sequence is highlighted in red. **B**. Exon and gene length of various *NUMB* homologs for which the genomic sequence is available; since most of the proteins are predicted from the genome, we calculated the length of each gene from the starting methionine to the stop codon of the protein. Exons coding for the PTB domain are in gray. (1) The *Takifugu rubripes* genome sequence of the *NUMB* locus has a gap in the assembly between Ex2 and Ex4 and therefore the length and presence of Ex3 is undetermined (N.A.: not available). (2) The *Petromyzon marinus* NUMB protein was predicted *ab initio* by the ENSEMBL pipeline with no support of any mRNA or EST, and the locus contains several gaps in the assembly: exons may be longer (+) or contain stop codons (*). Therefore, both the protein sequence and the genomic organization may not be accurate. (3) *Oikopleura dioica*, *Ciona intestinalis*, *Caenorhabditis elegans* and *Drosophila melanogaster* display a different genomic organization of the *NUMB* locus with respect to the vertebrate and other invertebrate loci, despite a very high similarity of the NUMB encoded proteins (Figure S2). (4) Ex4 (Ex5 with respect to the human gene) is not present in the *Hydra vulgaris* genome, possibly due to a gap in the assembly, thus exact length of flanking exons is not certain (N.A.: not available). (5) The first exon of the *Oscarella carmela NUMB* gene is incomplete, due to a gap in the assembly of the genome, and the genomic sequence of last exon(s) is not available (N.A.: not available). Exon phase numbers are reported between corresponding exons. **C**. Multiple alignment of the PTB domains of the various NUMB proteins. The alignment was manually adjusted to reflect the splicing position of invertebrate NUMB PTB domains between Ex2 and Ex4 (exon numbering is with respect to the human *NUMB* gene). Above the alignment, the secondary structure is depicted as determined by the 3D crystal structure, and residues involved in the binding to (p)Y-containing peptides are indicated by an asterisk (Colaluca *et al.* 2018). Splice site positions and exon phase within the human protein sequence are indicated by arrowheads. Gaps evidenced in red in the Oikopleura and Caenorhabditis proteins in the alignment, indicate the portion of the protein coded by Ex2 and Ex4 (with respect to the human gene) that is produced by a single exon (underlined in B). See also Figure S2 for the complete protein alignment.

All PTB domains encompass a minimal fold containing two orthogonally arranged β-sheets composed of seven anti-parallel β-strands. The β-sheets pack against two α-helices, α2 and α3 (Li *et al.* 1998, Uhlik, 2005 #38; Colaluca *et al.* 2018) (Figure 2A). The PTB domains of NUMB and NUMBL are well conserved from their first appearance in Ctenophora (46% and 48% identity between the Human and *Mnemiopsis leidyi* and *Pleurobrachia bachei* NUMB PTB domains, respectively), especially in the second half of the domain, encompassing strand β4 to helix α3. At the genomic level, this sequence is coded by Ex5 and Ex6 of the *NUMB* genes (Figure 2B). Moreover, strand β5 together with helix α3 (Figure 2C) plays a central role in the canonical binding properties to NPx(p)Y-containing peptides (Uhlik *et al.* 2005). Thus, it is not surprising that this region is the most conserved one in the domain. With the exception of *Caenorhabditis elegans*, *Drosophila melanogaster*, *Ciona intestinalis* and *Oikopleura dioica* (and possibly of Sea Lamprey, see below), the PTB domain of NUMB is coded by 6 or 5 exons, depending on the presence or absence of the exon corresponding to human Ex3 (Figure 2B).

Analysis of the PTB domain sequences of the various NUMB orthologs indicated that a PTB-long isoform first appeared in the chordate lineage, being present in *Branchiostoma* and *Ciona* (Cephalochordate and Urochordate/Tunicate) and absent in invertebrate proteins (Figure 2B, Figure S2). *NUMBL* orthologs do not encode for a PTB-long isoform (Figure S3) and there is no evidence supporting the existence of an alternative Ex3-like sequence in the genomes or transcriptomes analyzed.

Typically, the alternatively spliced Ex3 is represented by a short sequence of 33 nucleotides encoding for 11 amino acids (Figure 2B and Table 1). Exceptions were:

1. *Oikopleura dioica*, a larvacean tunicate similar to *Ciona intestinalis*, in which there are two distinct *NUMB-like* genes, both encoding a PTB-short-containing protein lacking the alternative exon. The *Oikopleura dioica NUMB* gene structure (GSOIDT00015583001) is remarkably different compared to other species (Figure 2B), and also with respect to the other *NUMB-Like* genes (GSOIDT00008916001) present in its genome (Figure S5). The two genes are very small (∼2 Kb), and Ex2 and Ex4 (numbering refers to the human gene) are fused in a single exon. These occurrences may not be surprising, since the genomes of tunicates exhibit great plasticity (Seo *et al.* 2001; Berná and Alvarez-Valin 2014) and the *Oikopleura dioica* genome was subject to massive intron loss (Denoeud *et al.* 2010). Of note, the *Oikopleura dioica* genome does not contain any P53- or MDM-related genes (data not shown).
2. *Ciona intestinalis* and *Branchiostoma floridae*, in which Ex3 (numbering refers to the human *NUMB* gene) is 36 and 39 nucleotides long, respectively (Figure 2B).
3. *Petromyzon marinus* (Sea Lamprey), representing the sole exception to the 33-nucleotide rule in vertebrates. The predicted NUMB protein displays a PTB insert of 14, rather than 11 amino acids, which - according to the ENSEMBL genome database - is coded by two short exons of 35 and 7 nucleotides, but only if the non-canonical splice site consensus is used in the protein prediction. If the canonical splice site rule (GT-AG) is employed, a similar exon organization of the *NUMB* gene is obtained (Figure 2B), but with two stop codons in the coding sequence. There are no mRNA sequences (or ESTs) to confirm the exact sequence of the transcript and thus of the protein. In addition, in this region of the genome there are several gaps in the assembly, thereby rendering it impossible to determine the reliability of this predicted protein sequence. Of note, the same predicted protein sequence and genomic organization (data not shown) are shared by the Japanese Lamprey (*Lethenteron camtschaticum*) (Figure S6).
4. *Takifugu rubripes*, in which the *NUMB* gene lacks Ex3, but this may be due to the presence of a gap in the genome assembly between Ex2 and Ex4. No Fugu mRNA or ESTs are available for *NUMB* (Table 1) to resolve this issue.

### The appearance of a NUMB PTB-long isoform is connected with the ability to bind and to regulate MDM2

Since the PTB-long isoform of NUMB first appeared in the chordate lineage, possibly in a common ancestor of Cephalochordates and Urochordates/Tunicates, we investigated whether in the tunicate, *Ciona intestinalis*, the different NUMB isoforms have evolved to display differential binding to MDM2 and possibly to regulate its functions, as occurs in human (Colaluca *et al.* 2018). We choose this organism since it belongs to the earliest branch in the Chordate phylum (Corbo *et al.* 2001) and the *MDM2* and *NUMB* cDNA are available (Satou *et al.* 2002; Karakostis *et al.* 2016).

**Figure 3:**
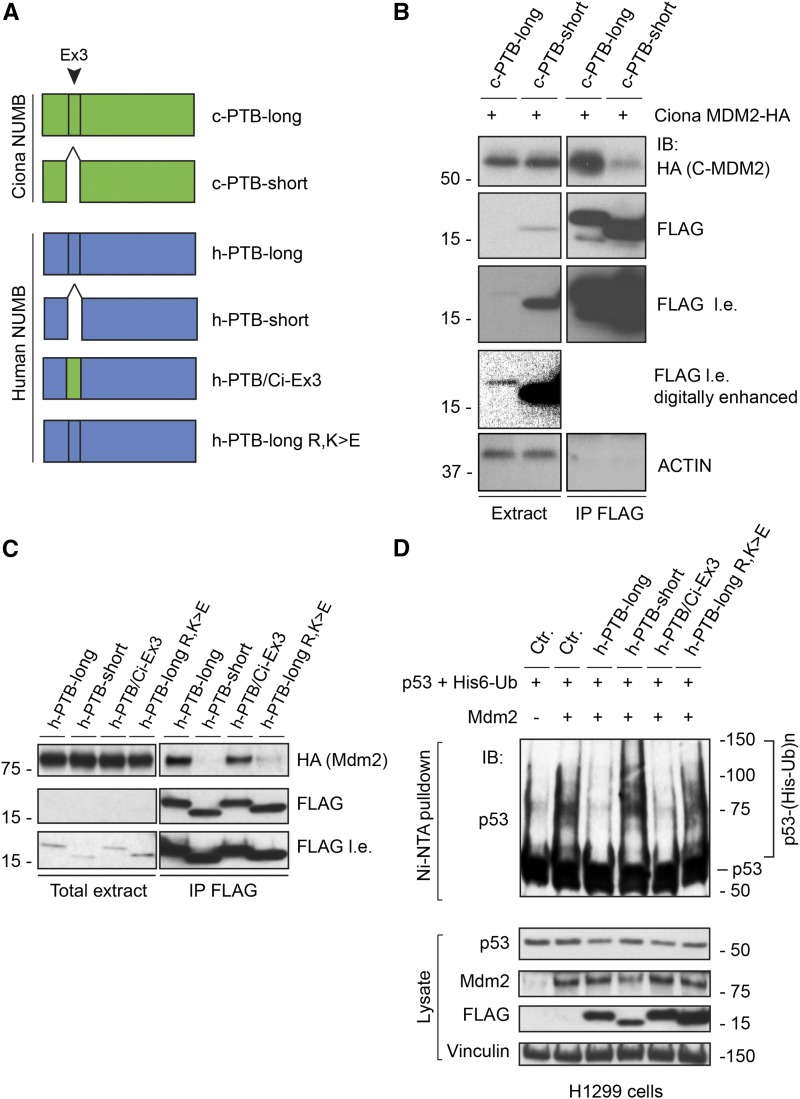
Ex3 of the *Ciona NUMB* gene can substitute for mammalian Ex3 to direct binding to MDM2 and regulation of P53. **A**. Schematic representation of the constructs used. c-PTB-long, *Ciona intestinalis* NUMB PTB long isoform; c-PTB-short, *Ciona intestinalis* NUMB PTB short isoform; h-PTB-long, human NUMB PTB long isoform; h-PTB-short, human NUMB PTB short isoform; h-PTB/Ci-Ex3, human NUMB short PTB isoform with the insertion of the Ciona Ex3 coded protein sequence; h-PTB-long R,K > E, human NUMB PTB long isoform with R69E-K70E-K73E-K78E substitutions (Colaluca *et al.* 2018). **B**. HEK293 cells were individually transfected with the indicated FLAG-tagged NUMB *Ciona* PTB (with or without the *Ciona* Ex3: c-PTB-long and c-PTB-short, respectively) and HA-tagged *Ciona* MDM2. IP (anti-FLAG) and IB were as shown. Intervening lanes were spliced out; l.e., long exposure. A digitally-enhanced image (curves tool in Photoshop CS6: shadow 152; highlight 165) of the anti-FLAG protein extracts is also shown. **C**. HEK293 cells were transfected with the indicated FLAG-tagged PTB constructs and HA-tagged human MDM2. IP (anti-FLAG) and IB were as shown (l.e., long exposure). **D**. MDM2-mediated P53 *in vivo* ubiquitination in H1299 cells transfected as indicated on the top. The Histidine-ubiquitinated proteins were purified as described in Materials and Methods and immunoblotted with the P53 antibody. The corresponding lysates were immunoblotted as indicated. A ratio Mdm2/p53 1.3:1 was used in these competition ubiquitination assays; at this protein ratio, only p53 ubiquitination and not its degradation is appreciable, as previously shown (Colaluca *et al.* 2018).

We transfected mammalian cells with constructs encoding one of the two versions of the *Ciona* NUMB PTB – Ex3-containing (c-PTB-long) and Ex3-devoid (c-PTB-short) (Figure 3A) – together with the *Ciona* MDM2. Only the c-PTB-long was able to coimmunoprecipitate efficiently with *Ciona* MDM2 (Figure 3B), suggesting that, since its initial appearance in evolution, the sequence coded by Ex3 confers to NUMB the ability to bind MDM2.

We then investigated whether the *Ciona* Ex3-coded sequence can substitute for the human Ex3-encoded sequence. To this end, we swapped the human Ex3 sequence for the *Ciona* one in the human NUMB PTB (Figure 3A). When expressed in HEK293 cells, this chimera (h-PTB/Ci-Ex3) coimmunoprecipitated with human MDM2 with an efficiency comparable to that of the WT human PTB-long (Figure 3C). In contrast, the human NUMB PTB-short and a human NUMB PTB-long mutant (PTB-long-R/K > E), previously engineered to abolish the interaction with MDM2 (Colaluca *et al.* 2018), were unable to coimmunoprecipitate with MDM2 (Figure 3C).

Next, we tested whether the *Ciona* Ex3 is also functionally equivalent to its human counterpart, by assessing the ability to inhibit MDM2-mediated P53 ubiquitination, as previously demonstrated (Colaluca *et al.* 2018). To this end, we set up an *in vivo* ubiquitination assay, by overexpressing in living H1299 cells, the human P53 and MDM2 proteins. Under these conditions, P53 polyubiquitination was readily detectable (compare the first two lanes of Figure 3D). As previously reported (Colaluca *et al.* 2018), the cotransfection of human PTB-long, but not of human PTB-short, reverted this effect (Figure 3D). Similarly, the cotransfection of the construct encoding the human/*Ciona* chimera, h-PTB/Ci-Ex3, inhibited the polyubiquitination of p53, to an extent comparable to that of human WT PTB-long (Figure 3D). We concluded that the function of the NUMB Ex3 sequence to bind and to inhibit the E3 ligase activity of MDM2 is conserved throughout evolution.

In summary, our findings show that Ex3 of NUMB, and hence the PTB-long isoforms of the protein, is a chordate invention. Since its initial appearance, which probably occurred in a common ancestor of Cephalochordates and Urochordates/Tunicates, Ex3 confers to NUMB the ability to bind and to regulate the E3 ligase activity of MDM2. MDM2 possesses in mammals many functions related to its protein:protein interaction abilities (Riley and Lozano 2012) and although the best studied function of MDM2 involves the direct control of p53, additional substrates of its E3 ligase activity exist, most of which however converge on the regulation of the p53 pathway (Riley and Lozano 2012). Thus, one appealing possibility is that the emergence of NUMB Ex3 may be directly connected to the regulation of the MDM2/P53 pathway in evolution. So far, few studies have explored the functions of MDM2 or P53 proteins in non-vertebrate organisms. The finding that in the ancient invertebrate metazoan, *Trichoplax adhaerens*, an MDM2-related protein can bind to *Trichoplax* P53 and induce its degradation (Siau *et al.* 2016), suggests that the signaling pathway of P53- and MDM-related protein families is ancient and co-evolved, although in some organisms, one or both players have been lost (Belyi *et al.* 2010; Momand *et al.* 2011; Åberg *et al.* 2017). In agreement with the current hypothesis of the evolution of the P53 protein family, the P53/63/73 ancestral function was that of monitoring the genetic quality in germline cells, as has been shown in *Caenorhabditis elegans* (Derry *et al.* 2001), in the sea anemone *Nematostella vectensis* (Pankow and Bamberger 2007) and in *Ciona intestinalis* (Noda 2011). Later in evolution, P63 and P73 preserved the ancestral functions in the development of tissues and organs (Levrero *et al.* 2000), becoming partly independent from MDM2 with respect to ubiquitination and degradation (Bálint *et al.* 1999; Zeng *et al.* 1999; Little and Jochemsen 2001; Kadakia *et al.* 2001; Galli *et al.* 2010; Armstrong *et al.* 2016), whereas P53 developed new functions, becoming the guardian of the somatic genome and a tumor suppressor, with MDM2 acting as its primary “director” (Belyi *et al.* 2010; Åberg *et al.* 2017). The emergence of a novel NUMB splicing isoform as a partner and regulator of MDM2 in Chordata, suggests a specific role for NUMB in the regulation of MDM2 signaling pathways, which in higher Metazoa culminate in the fine-tuning of P53 activity in somatic tissues.
